# Short-term exposure of PM_2.5_ and PM_10_ increases the number of outpatients with eczema in Guangzhou: A time-series study

**DOI:** 10.3389/fpubh.2022.930545

**Published:** 2023-02-21

**Authors:** Ji Zhang, Yicheng Yang, Lin Fu, Dian Jing, Bo Sun, Yilin Chen, Junyi Chen, Shuqun Shen

**Affiliations:** ^1^Zhujiang Hospital, Southern Medical University, Guangzhou, Guangdong, China; ^2^State Key Laboratory of Cardiovascular Disease, Fuwai Hospital, National Center for Cardiovascular Diseases, Chinese Academy of Medical Sciences and Peking Union Medical College, Beijing, China; ^3^Graduate School of Peking Union Medical Chinese Academy of Medical Sciences Plastic Surgery Hospital, Beijing, China; ^4^Department of Public Health, Southern Medical University, Guangzhou, China; ^5^Department of Cardiology, Guangdong Cardiovascular Institute, Guangdong Provincial People's Hospital, Guangdong Academy of Medical Sciences, School of Medicine, South China University of Technology, Guangzhou, China; ^6^Dermatology Hospital, Southern Medical University, Guangzhou, China

**Keywords:** short-term exposure, particulate matter, PM_2.5_, PM_10_, air pollution, eczema, outpatients

## Abstract

**Background:**

The worldwide prevalence of eczema has continued to rise over the past decades. This has led to the emphasis on the association between air pollution and eczema. This study investigated the relationship between daily exposure to air pollution and the number of eczema outpatient visits in Guangzhou with the overarching goal of providing novel insights on the interventions for eczema aggravation and prevention.

**Methods:**

Daily air pollution data, meteorological data, and the number of eczema outpatients were obtained from 18 January 2013 to 31 December 2018 in Guangzhou. A generalized additive model with Poisson distribution was used to assess the association between the number of eczema outpatient visits and short-term exposure to PM_2.5_ and PM_10_. In addition, the association of PM_2.5_ and PM_10_ by age (<65 years, ≥65 years) and gender was evaluated.

**Results:**

A total of 293,343 eczema outpatient visits were recorded. The obtained results indicated that a 10 μg/m^3^ increase of the same day/lag 1 day/lag 2 days PM_2.5_ was associated with increments of 2.33%, 1.81%, and 0.95% in eczema outpatient risk, respectively. On the other hand, a 10 μg/m^3^ increase of PM_10_ was associated with eczema outpatients risk increments of 1.97%, 1.65%, and 0.98% respectively. Furthermore, the associations of PM on the increment of eczema were similar in the male and female groups. Results obtained after age stratified analyses indicated that the strongest positive association between PM_2.5_ exposure and eczema was observed at lag 0 day with the percent changes being 4.72% and 3.34% in <12 years old, ≥12 and <65 years old, and ≥65 years old groups, respectively.

**Conclusion:**

Short-term exposure to PM_2.5_ and PM_10_ increases the number of eczema outpatients, especially among children and the elderly. The relationship between air quality trends and hospital resource arrangement should be paid attention to by hospital managers which may aid in disease prevention and lower the health burden.

## Background

An eczema is a group of common inflammatory skin diseases. Patients may develop different skin lesions and relapsing pruritus in the different stages, thereby causing a considerable physical and psychological burden on the patients (1–3). The worldwide prevalence of eczema has been rising continuously over the past decades (4, 5). The available clinical treatments only improve the symptoms as opposed to curing the disease (5). Therefore, the lack of a cure for eczema highlights the importance of prevention. A previous study has reported that the identification of the underlying modifiable risk factors for eczema including environmental stimuli plays a decisive role in the prophylactic management of the disease (6).

In addition to the genetic background, environment, dietary habits, lifestyle, and societal status have also been associated with the pathogenic condition of skin diseases (7, 8). Air pollution, including particulate matter (PM), nitrogen dioxide (NO_2_), and sulfur dioxide (SO_2_), is one of the environmental stimuli (9). Recently, our team has revealed the tight association between exposure to NO_2_ with eczema incidence and outpatient visits (10). In addition to NO_2_, accumulating scientific evidence has reported the associations of PM with the development of eczema and dermatitis (11, 12). PM is naturally produced from salt and dust while the majority of PM is derived from anthropogenic activities of fossil fuel combustion, mostly contributed by the consumption of fuel by vehicles (13). PM is a complicated mixture with alternative concentrations, particle diameter, and chemical properties in various spaces and times (14). According to the particles' aerodynamic diameter, PM can be categorized into PM_0.1_ (particle diameter ≤ 0.1 μm), PM_2.5_ (particle diameter ≤ 2.5 μm), and PM_10_ (particle diameter ≤ 10 μm) (15).

Recently, the article revealed the aggravation of PM in children's atopic dermatitis in South Korea but did not mention adult and elderly patients (16). The skin manifestations of patients with AD of different ages are different. Therefore, we studied the effects of PM on dermatitis in children, adults, and the elderly to reveal the effect of PM on patients with AD in different age categories is consistent. We expect PM to have similar effects in children as in previous studies, as well as in the elderly. Some studies showed PM enables to inducement of skin inflammation and deteriorates inflammatory skin disorder symptoms, which may attribute to the mechanisms of oxidative stress or programmed cell death. However, the exact and complete mechanisms of skin disease induced by PM are still calling for exploration.

Moreover, a meta-analysis (17) included 13 clinical studies, a total of 72,000 participants, aiming to demonstrate the effects of PM_2.5_ and PM_10_ on human skin diseases including atopic dermatitis, eczema, and skin aging. Short-term effects have been estimated that increases of 10 μg/m^3^ in PM_2.5_ and PM_10_ were associated with a 1.60% (0.45–2.82) and 1.01% (0.08–2.05) increase in total skin disease risk, respectively. In addition, the study has demonstrated that long-term exposure to PM_2.5_ upwards of 47.09 μg/m^3^ and PM_10_ upwards of 26.04 μg/m^3^ would adversely affect human skin. However, this study failed to reveal the effect of PM on eczema specially and up to now, evidence of the association between PM and eczema is still lacking.

Guangzhou, with a population density of 2,059 people/km^2^ is the 3rd most densely populated city in China in 2019 (https://www.askci.com/news/data/hongguan/20200730/1441261166838.shtml). A previous study reported that PM has become one of the main sources of air pollution in Guangzhou due to rapid industrial development and advanced transportation (18). Therefore, this study investigated the association between daily air pollution exposure and the number of outpatients with eczema in Guangzhou. In addition, the previously mentioned associations were also evaluated in different ages, and gender subgroups. The main aim of the study was to identify the potential environmental risk factors and to provide novel insights into the interventions for eczema aggravation and prevention.

## Materials and methods

### Study setting

Guangzhou, the capital city of Guangdong province, is one of the developed cities in China with an approximate population of 14.9 million in 2018. The city, located at 23°10′N, 113°18′E, has a typical subtropical humid-monsoon climate with an average annual temperature and rainfall of 22°C and 1,500–2,000 mm, respectively (http://tjj.gz.gov.cn/tjdt/content/post_5727607.html). In addition, Guangzhou is the top-ranking commercial and manufacturing city in China. Therefore, the problem of air pollution in the city as a result of economic advancement should not be underestimated. In 2019, the average concentrations of PM_2.5_, PM_10_, NO_2_, SO_2_, and O_3_ in Guangzhou were 30 ug/m^3^, 53 ug/m^3^, 45 ug/m^3^, 7 ug/m^3^, and 178 ug/m^3^, respectively^1^, while in Haikou, China, the average concentrations were lower as 17 ug/m^3^, 32 ug/m^3^, 13 ug/m^3^, 5 ug/m^3^, and 144 ug/m^3^ respectively (http://www.haikou.gov.cn/xxgk/szfbjxxgk/tjxx/hjzk/202006/t20200630_1519119.html).

### Data sources

#### Patient data

The Dermatology Hospital of Southern Medical University, located in Tianhe district, Guangzhou, is a large-scale dermatology specialty and third-class hospital in South China. The hospital has the largest number of eczema outpatient visits in Guangzhou (http://www.gdskin.com/ShowClass.aspx?ID=704). This study enrolled outpatients who were diagnosed with eczema coded as L30.902 according to the World Health Organization's International Classification of Diseases [the 10th version (ICD-10)] in the hospital from 18 January 2013 to 31 December 2018. If recurrent eczema occurred within 21 days of a previous outpatient visit, it was regarded as a single eczema event.

#### Air pollution and meteorological data

Air quality monitoring data was obtained from the public sharing system of the Guangzhou Environmental Monitoring Center (http://sthjj.gz.gov.cn/infoindex.html). Data recorded in the system was obtained from 11 national air quality monitor stations in Guangzhou and the daily average air quality data was used for our analyses. Daily average concentrations of PM_2.5_, PM_10_, NO_2_, SO_2_, and O_3_ during the period ranging from 18 January 2013 to 31 December 2018 were also collected. Daily meteorological data, including mean humidity and temperature, were obtained from the China Meteorological Data Sharing Service System based on data from different meteorological stations in Guangzhou and used for analyses. Both air pollution and meteorology data followed the quality control programs which are mandated by the Chinese government.

### Statistical analyses

A generalized additive model (GAM) with Poisson distribution was used to assess the association between eczema outpatient visits and short-term exposure to PM_2.5_ and PM_10_ (19). Lag 0–3 days were used to explore the cumulative exposure and displacement associations of PM and other pollutants (20). This study used a single pollutant model to examine the association between PM and eczema admission. In addition, we built a two-pollutant model to control the potential confounding effects of other pollutants (21). These models can be represented as follows:


$$\begin{array}{l} log\left(\mu_{t}\right)=\alpha +\sum \beta_{i}X_{it}+NS\left({temperature}_{t},df=3\right)\\ +NS\left({humidity}_{t},df=3\right)+NS\left({time}_{t},df=10/year\right)+\gamma {DOW}_{t} \end{array}$$

where μ_*t*_ represents the expected number of eczema outpatients on day *t*; α is the intercept; *X*_*it*_ represents the concentrations of pollutants (PM_2.5_, PM_10_, NO_2_, SO_2_, and O_3_) on day *t, i* = 1 or *i* = 2 represents single pollutant model or two-pollutant model, respectively; and β_*i*_ stands for the coefficient of *X*_*i*_. A natural cubic spline function (NS) with 10, 3, and 3 degrees of freedom (df) was used to capture the non-linear relationships of time trends, temperature, and humidity (22). In our model, the day of week (*DOW*_*t*_) was set in the form of categorical variables, while γ represents the effect of *DOW*_*t*_ on eczema outpatients.

Age and gender stratified analyses using potential individual-level effect modifiers were also conducted. This study used the above basic models to examine outcomes by stratification of age (<65 years, ≥65 years) and gender for the purpose of exploring the potential modification.

In addition, the dfs for temperature, humidity, and time used in the above models were assessed using the Akaike information criterion for quasi-Poisson (Q-AIC). The minimum value of Q-AIC represented the best goodness and the optimum of dfs. In order to check the robustness of our modeling strategies, sensitivity analyses were done by changing the df for temperature (2–4), humidity (2–4), and calendar time (9–11 per year) to control time trends (23). R software version 4.0.2 was used to conduct all the analyses and a *P* < 0.05 was considered to be statistically significant in all statistical analyses.

## Results

### Data description

**Figure 1:**
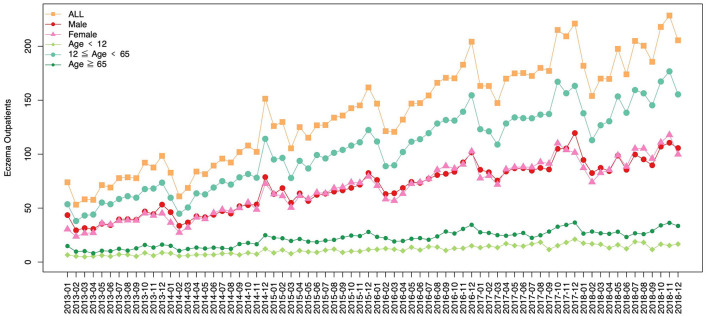
A line plot presenting the time trend of eczema events (monthly average) overall and by sex and age groups.

In this study, a total of 293,343 eczema outpatients were recorded in the Dermatology Hospital of Southern Medical University from 18 January 2013 to 31 December 2018. Table 1 shows the descriptive statistics of daily eczema outpatients, air pollutants, and meteorological variables. The average number of daily eczema outpatients in the hospital was 190 in 2018, while the average concentrations of air pollutants including PM_2.5_, PM_10_, NO_2_, SO_2_, and O_3_ were 34.7 μg/m^3^, 55.7 μg/m^3^, 48.1 μg/m^3^, 9.6 μg/m^3^, and 50.7 μg/m^3^, respectively. In addition, the annual mean temperature and humidity were 22.3°C and 81.7%, respectively. With the exception of temperature (*F* value = 0.313, *P* = 0.905), the annual average values of all other variables were significantly different from 2013 to 2018. Furthermore, there was an increased trend of eczema outpatient visits. Figure 1 is presenting the time trend of eczema events overall and by sex and age groups during the study period.

**Table 1:** Daily outpatients of eczema, meteorological factors and air pollutants in Guangzhou (2013/1/18–2018/12/31).

	**2013**	**2014**	**2015**	**2016**	**2017**	**2018**	***F***	***P***
Eczema outpatients	75.261 ± 23.840	93.688 ± 31.839	131.406 ± 37.233	155.644 ± 43.374	180.638 ± 48.581	190.845 ± 48.430	478.389	<0.001
Temperature^a^ (°C)	22.160 ± 5.585	21.768 ± 6.580	22.229 ± 5.927	22.019 ± 6.395	22.136 ± 5.837	22.255 ± 6.177	0.313	0.905
Humidity^*^ (%)	81.296 ± 11.546	78.448 ± 10.628	78.057 ± 8.907	81.991 ± 9.698	80.942 ± 11.373	81.685 ± 10.203	9.472	<0.001
${\text{PM}}_{2.5}^{*}$ (mg/m^3^)	50.165 ± 27.241	49.350 ± 24.731	38.958 ± 21.112	34.163 ± 17.220	35.033 ± 19.055	34.663 ± 21.135	40.575	<0.001
${\text{PM}}_{10}^{*}$ (mg/m^3^)	72.139 ± 34.403	67.476 ± 30.655	60.636 ± 28.122	54.739 ± 25.081	56.388 ± 27.007	55.658 ± 27.538	21.298	<0.001
${\text{NO}}_{2}^{*}$ (mg/m^3^)	51.237 ± 21.476	44.093 ± 18.826	45.188 ± 17.063	43.716 ± 18.109	49.664 ± 19.595	48.064 ± 19.605	9.315	<0.001
${\text{SO}}_{2}^{*}$ (mg/m^3^)	20.798 ± 8.447	20.953 ± 15.474	12.998 ± 5.216	11.954 ± 3.783	11.739 ± 3.645	9.603 ± 3.262	135.621	<0.001
${\text{O}}_{3}^{*}$ (mg/m^3^)	61.797 ± 36.736	57.796 ± 33.119	47.715 ± 24.465	47.175 ± 24.994	50.253 ± 27.124	50.678 ± 25.623	14.398	<0.001

a F = 0.313, P = 0.905.

* P <0.001.

Obtained results indicated that the correlation coefficients between PM_2.5_ and temperature, humidity, PM_10_, NO_2_, SO_2_, and O_3_ were 0.32, 0.22, 0.94, 0.73, 0.60, and 0.31, respectively. PM_2.5_ was negatively correlated with temperature and humidity and positively correlated with PM_10_, NO_2_, SO_2_, and O_3_. On the other hand, the correlation coefficients between PM_10_ and temperature, humidity, PM_2.5_, NO_2_, SO_2_, and O_3_ were 0.24, 0.26, 0.94, 0.80, 0.44, and 0.35, respectively. Similarly, PM_10_ was negatively correlated with temperature and humidity and positively correlated with PM_2.5_, NO_2_, SO_2_, and O_3_. The highest Pearson correlations were observed between PM_2.5_ and PM_10_ in Table 2.

**Table 2:** The correlation coefficients between PM_2.5_, PM_10_, and other pollutants.

	**Temperature**	**Humidity**	**PM_2.5_**	**O_3_**	**PM_10_**	**SO_2_**	**NO_2_**
Temperature	–	0.31^**^	−0.32^**^	0.20^**^	−0.24^**^	−0.15^**^	−0.27^**^
Humidity	–	–	−0.22^**^	−0.41^**^	−0.26^**^	−0.17^**^	0.02
PM_2.5_	–	–	–	0.31^**^	0.94^**^	0.60^**^	0.73^**^
O_3_	–	–	–	–	0.35^**^	0.26^**^	0.11^**^
PM_10_	–	–	–	–	–	0.44^**^	0.80^**^
SO_2_	–	–	–	–	–	–	0.27^**^

** P < 0.0001.

### Distributed lag non-linear model analyses

The smallest Q-AIC of 27,558.6 was obtained with per year df of 3, 3, 10 for temperature, humidity, and time, respectively, in Table 3. This result formed the basis for the distributing lag non-linear model analyses which followed.

**Table 3:** Results of Akaike information criterion for quasi-Poisson (Q-AIC).

**Time, DF (/year)**	**Temperature, DF**	**Humidity, DF**							
		**3**	**4**	**5**	**6**	**7**	**8**	**9**	**10**
8	3	27„687.49	27,698.63	27„710.13	27,713.52	27„720.51	27,730.71	27,733.56	27,735.89
	4	27,697.89	27,709.01	27,720.50	27,723.89	27,730.41	27,740.75	27,743.74	27,745.83
	5	27,705.25	27,716.44	27,727.74	27,731.32	27,737.59	27,747.87	27,750.27	27,752.44
	6	27,714.62	27,725.84	27,737.11	27,740.58	27,746.86	27,757.19	27,759.62	27,761.81
	7	27,718.72	27,729.97	27,741.24	27,744.36	27,750.68	27,761.08	27,763.70	27,766.01
	8	27,694.57	27,705.30	27,716.62	27,719.05	27,725.94	27,736.22	27,738.77	27,742.45
	9	27,695.71	27,706.24	27,717.44	27,719.90	27,726.16	27,737.02	27,739.44	27,742.62
	10	27,701.47	27,711.98	27,722.74	27,725.05	27,731.92	27,743.25	27,745.65	27,748.14
9	3	27,650.09	27,661.39	27,672.00	27,673.68	27,679.41	27,688.31	27,692.51	27,695.53
	4	27,661.41	27,672.71	27,683.41	27,685.04	27,690.90	27,699.79	27,704.04	27,707.11
	5	27,669.24	27,680.59	27,691.06	27,692.94	27,698.59	27,707.45	27,711.32	27,714.42
	6	27,678.54	27,689.91	27,700.32	27,702.12	27,707.80	27,716.70	27,720.56	27,723.69
	7	27,683.88	27,695.26	27,705.69	27,707.15	27,712.83	27,721.83	27,725.83	27,729.02
	8	27,661.06	27,672.12	27,682.52	27,683.34	27,689.60	27,698.53	27,702.70	27,707.03
	9	27,663.26	27,674.19	27,684.39	27,685.27	27,690.84	27,700.41	27,704.45	27,708.37
	10	27,669.28	27,680.20	27,689.80	27,690.48	27,696.72	27,706.81	27,710.80	27,714.25
10	3	27,558.60	27,570.00	27,579.86	27,582.72	27,586.32	27,595.13	27,599.11	27,601.70
	4	27,564.76	27,576.08	27,586.21	27,589.03	27,593.53	27,602.16	27,606.28	27,609.24
	5	27,570.71	27,582.11	27,591.93	27,594.91	27,599.01	27,607.66	27,611.29	27,614.29
	6	27,579.87	27,591.27	27,600.96	27,603.89	27,608.17	27,616.79	27,620.38	27,623.46
	7	27,587.79	27,599.20	27,608.96	27,611.69	27,615.91	27,624.67	27,628.44	27,631.55
	8	27,570.61	27,581.98	27,591.66	27,593.79	27,598.67	27,607.49	27,611.45	27,615.53
	9	27,573.03	27,584.35	27,593.81	27,595.89	27,600.09	27,609.48	27,613.28	27,617.01
	10	27,579.26	27,590.61	27,599.42	27,601.30	27,606.12	27,615.98	27,619.68	27,622.92

Results obtained from the single pollutant model indicated that a per 10 g/m^3^ increase of PM_2.5_ was associated with a 2.33% (95%CI: 2.06%, 2.60%, *P* < 0.001), 1.81% (95%CI: 1.53%, 2.09%, *P* < 0.001), 0.95% (95%CI: 0.68%, 1.23%, *P* < 0.001), and 0.29% (95%CI: 0.29%, 0.56%, *P* = 0.274) increase in the number of daily eczema outpatients at lag 0–3 days, respectively. In addition, a per 10 g/m^3^ increase of PM_10_ was interrelated to a 1.97% (95%CI: 1.77%, 2.17%, *P* < 0.001), 1.65% (95%CI: 1.45%, 1.86%, *P* < 0.001), 0.98% (95%CI: 0.78%, 1.18%, *P* < 0.001), and 0.52% (95%CI: 0.32%, 0.71%, *P* = 0.008) increase in the number of daily eczema outpatients at lag 0–3 days, respectively, in Table 4.

**Table 4:** The RR and percent change of eczema risk associated with 10 mg/m^3^ increase of PM_2.5_ and PM_10_.

**Pollutant (per 10 mg/m^3^)**	**Lag day**	**RR**	**PC (%)**	***P***
PM_2.5_	Lag 0	1.0233 (1.0206–1.0260)	2.33 (2.06–2.6)	<0.001
	Lag 1	1.0181 (1.0153–1.0209)	1.81 (1.53–2.09)	<0.001
	Lag 2	1.0095 (1.0068–1.0123)	0.95 (0.68–1.23)	<0.001
	Lag 3	1.0029 (1.0003–1.0056)	0.29 (0.03–0.56)	0.274
PM_10_	Lag 0	1.0197 (1.0177–1.0217)	1.97 (1.77–2.17)	<0.001
	Lag 1	1.0165 (1.0145–1.0186)	1.65 (1.45–1.86)	<0.001
	Lag 2	1.0098 (1.0078–1.0118)	0.98 (0.78–1.18)	<0.001
	Lag 3	1.0052 (1.0032–1.0071)	0.52 (0.32–0.71)	0.008

RR, risk ratio; PC, percentage change.

On the other hand, results obtained from the two-pollutant model analyses indicated that a per 10 g/m^3^ increase of PM_2.5_ adjusting for PM_10_, NO_2_, SO_2_, and O_3_ was related to a 1.29% (95%CI: 1.13%, 1.45%), 2.01% (95%CI: 1.80%, 2.23%), 2.54% (95%CI: 2.23%, 2.85%), and 1.46% (95%CI: 1.29%, 1.60%) increase of eczema outpatient visits at lag 0–3 days, respectively. In addition, per 10 g/m^3^ increase of PM_10_ increased eczema outpatient visits by 1.75% (95%CI: 1.58%, 1.92%), 2.34% (95%CI: 2.10%, 2.58%), and 1.4% (95%CI: 1.25%, 1.54%) at lag 0–3 days, respectively, after adjusting for NO_2_, SO_2_, and O_3_ in Table 5.

**Table 5:** The RR and percent change of eczema risk associated with 10 mg/m^3^ increase of PM_2.5_ and PM_10_ under two-pollutant model.

**Pollutant (per 10 mg/m^3^)**	**Model type**	**RR**	**PC (%)**
${\text{PM}}_{2.5}^{*}$	PM_2.5_ + PM_10_	1.0129 (1.0113–1.0145)	1.29 (1.13–1.45)
	PM_2.5_ + NO_2_	1.0201 (1.018–1.0223)	2.01 (1.8–2.23)
	PM_2.5_ + SO_2_	1.0254 (1.0223–1.0285)	2.54 (2.23–2.85)
	PM_2.5_ + O_3_	1.0146 (1.0129–1.0163)	1.46 (1.29–1.63)
${\text{PM}}_{10}^{*}$	PM_10_ + NO_2_	1.0175 (1.0158–1.0192)	1.75 (1.58–1.92)
	PM_10_ + SO_2_	1.0234 (1.021–1.0258)	2.34 (2.1–2.58)
	PM_10_ + O_3_	1.014 (1.0125–1.0154)	1.4 (1.25–1.54)

^*^P < 0.001.

RR, risk ratio; PC, percentage change.

### Stratified analyses

Results obtained after gender stratified analyses indicated that both in the male and female group, a 10 μg/m^3^ increase of PM_2.5_ or PM_10_ concentrations was associated with an increased risk of eczema outpatient visits at lag 0–3 days, respectively. Gradual weakening trends could also be observed at a lag of 1–3 days in Table 6. Both in the male and female groups, the strongest associations between PM_2.5_ [2.32%, (95%CI: 2.03%, 2.61%, *P* < 0.001) vs. 2.31% (95%CI: 2.01%, 2.61%, *P* < 0.001)] and PM_10_ [1.97% (95%CI: 1.76%, 2.18%, *P* < 0.001) vs. 1.97%, (95%CI: 1.75%, 2.19%, *P* < 0.001)] on lag 0 were figured out. Age stratified analyses results indicated that the strongest positive association between PM_2.5_ or PM_10_ exposure and eczema were also observed at lag 0 day with the percent changes being [4.72% (95%CI, 4.18%, 5.28%, *P* < 0.001) vs. 3.74% (95%CI, 3.34%, 4.14%, *P* < 0.001)], [1.92% (95%CI: 1.65%, 2.19%, *P* < 0.001) vs. 1.66% (95%CI, 1.47%, 1.86%, *P* < 0.001)] and [3.34% (95%CI: 2.90%, 3.78%, *P* < 0.001) vs. 2.55%, (95%CI: 2.24%, 2.87%, *P* < 0.001)] in the <12 years old, ≥12 and <65 years old, and ≥65 years old groups, respectively. Moreover, Table 7 showed the similar associations of PM_2.5_ and PM_10_ on the number of eczema outpatients in both the two-pollutant model and the single pollutant model.

**Table 6:** Stratification analysis of the percent change of eczema risk associated with 10 mg/m^3^ increase of PM_2.5_ and PM_10_.

**Pollutant (per 10 mg/m^3^)**	**Lag day**	**Male**		**Female**		<**12**		**12–64**		≥**65**	
		**PC (%)**	***P***	**PC (%)**	***P***	**PC (%)**	***P***	**PC (%)**	***P***	**PC (%)**	***P***
PM_2.5_	Lag 0	2.32 (2.03–2.61)	<0.001	2.31 (2.01–2.61)	<0.001	4.72 (4.18–5.28)	<0.001	1.92 (1.65–2.19)	<0.001	3.34 (2.9–3.78)	<0.001
	Lag 1	1.77 (1.47–2.07)	<0.001	1.82 (1.51–2.13)	<0.001	3.11 (2.54–3.68)	<0.001	1.59 (1.31–1.87)	<0.001	2.27 (1.82–2.73)	<0.001
	Lag 2	0.9 (0.61–1.19)	0.002	0.99 (0.68–1.29)	0.001	0.91 (0.36–1.46)	0.096	0.84 (0.57–1.12)	0.002	1.59 (1.14–2.03)	<0.001
	Lag 3	0.16 (-0.13–0.44)	0.579	0.41 (0.12–0.71)	0.160	−0.74 (−1.26-−0.22)	0.159	0.3 (0.03–0.56)	0.26	1.15 (0.72–1.59)	0.008
PM_10_	Lag 0	1.97 (1.76–2.18)	<0.001	1.97 (1.75–2.19)	<0.001	3.74 (3.34–4.14)	<0.001	1.66 (1.47–1.86)	<0.001	2.55 (2.24–2.87)	<0.001
	Lag 1	1.6 (1.38–1.81)	<0.001	1.71 (1.48–1.93)	<0.001	2.81 (2.4–3.23)	<0.001	1.46 (1.26–1.66)	<0.001	1.9 (1.57–2.23)	<0.001
	Lag 2	0.94 (0.73–1.15)	<0.001	1.01 (0.79–1.23)	<0.001	1.16 (0.77–1.56)	0.003	0.88 (0.69–1.08)	<0.001	1.27 (0.95–1.59)	<0.001
	Lag 3	0.39 (0.18–0.6)	0.059	0.65 (0.43–0.86)	0.002	−0.27 (-0.64–0.12)	0.485	0.53 (0.33–0.72)	0.006	0.99 (0.68–1.3)	0.002

PC, percentage change.

**Table 7:** Stratification analysis of the percent change of eczema risk associated with 10 mg/m^3^ increase of PM_2.5_ and PM_10_ under two-pollutant model.

**Pollutant (per 10 mg/m^3^)**	**Model type**	**Male**	**Female**	** <12**	**12–64**	**≥65**
${\text{PM}}_{2.5}^{*}$ PM10^*^	PM_2.5_ + PM_10_	1.24 (1.06–1.41)	1.34 (1.17–1.52)	1.86 (1.54–2.18)	1.13 (0.97–1.3)	1.81 (1.55–2.07)
	PM_2.5_ + NO_2_	1.96 (1.73–2.19)	2.04 (1.81–2.28)	2.65 (2.22–3.08)	1.79 (1.58–2.01)	2.67 (2.32–3.02)
	PM_2.5_ + SO_2_	2.46 (2.12–2.79)	2.6 (2.25–2.94)	3.71 (3.09–4.34)	2.22 (1.91–2.54)	3.65 (3.15–4.16)
	PM_2.5_ + O_3_	1.41 (1.22–1.59)	1.52 (1.33–1.71)	2.55 (2.2–2.89)	1.26 (1.09–1.43)	2.02 (1.74–2.3)
	PM_2.5_ + NO_2_	1.69 (1.51–1.88)	1.8 (1.61–1.99)	2.35 (2–2.7)	1.57 (1.4–1.74)	2.19 (1.91–2.47)
	PM_2.5_ + SO_2_	2.25 (2–2.51)	2.42 (2.15–2.68)	3.38 (2.9–3.87)	2.07 (1.84–2.31)	3.03 (2.64–3.41)
	PM_2.5_ + O_3_	1.34 (1.19–1.5)	1.46 (1.3–1.62)	2.33 (2.03–2.62)	1.22 (1.07–1.37)	1.82 (1.58–2.06)

^*^P < 0.001.

### Sensitivity analyses

The obtained results after sensitivity analyses done in this study showed that the association estimates of PM_2.5_ and PM_10_ were not materially influenced by changing the df for the smooth function of the time trend indicating the credibility of our results in Table 8.

**Table 8:** The RR and percent change of eczema risk associated with 10 mg/m^3^ increase of PM_2.5_ and PM_10_ with different degrees of freedom.

**Temperature, DF**	**Humidity, DF**	**Time, DF (/year)**	**PM**_**2.5**_		**PM**_**10**_	
			**RR** ^*^	**PC (%)** ^*^	**RR** ^*^	**PC (%)** ^*^
2	2	9	1.026 (1.0222–1.0298)	2.6 (2.22–2.98)	1.0238 (1.0211–1.0265)	2.38 (2.11–2.65)
2	2	10	1.0257 (1.0218–1.0295)	2.57 (2.18–2.95)	1.0238 (1.0211–1.0266)	2.38 (2.11–2.66)
2	2	11	1.0246 (1.0207–1.0285)	2.46 (2.07–2.85)	1.0234 (1.0206–1.0262)	2.34 (2.06–2.62)
2	3	9	1.026 (1.0222–1.0298)	2.6 (2.22–2.98)	1.0238 (1.0211–1.0265)	2.38 (2.11–2.65)
2	3	10	1.0257 (1.0218–1.0295)	2.57 (2.18–2.95)	1.0238 (1.021–1.0265)	2.38 (2.1–2.65)
2	3	11	1.0246 (1.0207–1.0285)	2.46 (2.07–2.85)	1.0234 (1.0206–1.0262)	2.34 (2.06–2.62)
2	4	9	1.026 (1.0222–1.0299)	2.6 (2.22–2.99)	1.0238 (1.0211–1.0265)	2.38 (2.11–2.65)
2	4	10	1.0257 (1.0218–1.0295)	2.57 (2.18–2.95)	1.0238 (1.0211–1.0265)	2.38 (2.11–2.65)
2	4	11	1.0246 (1.0207–1.0285)	2.46 (2.07–2.85)	1.0233 (1.0205–1.0262)	2.33 (2.05–2.62)
3	2	9	1.0262 (1.0224–1.0301)	2.62 (2.24–3.01)	1.024 (1.0213–1.0267)	2.4 (2.13–2.67)
3	2	10	1.0259 (1.0221–1.0298)	2.59 (2.21–2.98)	1.0241 (1.0213–1.0268)	2.41 (2.13–2.68)
3	2	11	1.0249 (1.021–1.0288)	2.49 (2.1–2.88)	1.0237 (1.0209–1.0265)	2.37 (2.09–2.65)
3	3	9	1.0262 (1.0224–1.0301)	2.62 (2.24–3.01)	1.024 (1.0213–1.0267)	2.4 (2.13–2.67)
3	3	10	1.0259 (1.022–1.0298)	2.59 (2.2–2.98)	1.0241 (1.0213–1.0268)	2.41 (2.13–2.68)
3	3	11	1.0248 (1.0209–1.0288)	2.48 (2.09–2.88)	1.0236 (1.0208–1.0264)	2.36 (2.08–2.64)
3	4	9	1.0262 (1.0224–1.0301)	2.62 (2.24–3.01)	1.024 (1.0213–1.0267)	2.4 (2.13–2.67)
3	4	10	1.0259 (1.022–1.0298)	2.59 (2.2–2.98)	1.0241 (1.0213–1.0268)	2.41 (2.13–2.68)
3	4	11	1.0248 (1.0209–1.0288)	2.48 (2.09–2.88)	1.0236 (1.0208–1.0264)	2.36 (2.08–2.64)
4	2	9	1.0264 (1.0226–1.0303)	2.64 (2.26–3.03)	1.0242 (1.0214–1.0269)	2.42 (2.14–2.69)
4	2	10	1.0264 (1.0225–1.0303)	2.64 (2.25–3.03)	1.0244 (1.0217–1.0272)	2.44 (2.17–2.72)
4	2	11	1.0254 (1.0214–1.0293)	2.54 (2.14–2.93)	1.024 (1.0212–1.0268)	2.4 (2.12–2.68)
4	3	9	1.0264 (1.0225–1.0302)	2.64 (2.25–3.02)	1.0242 (1.0214–1.0269)	2.42 (2.14–2.69)
4	3	10	1.0263 (1.0225–1.0302)	2.63 (2.25–3.02)	1.0244 (1.0216–1.0272)	2.44 (2.16–2.72)
4	3	11	1.0253 (1.0214–1.0293)	2.53 (2.14–2.93)	1.024 (1.0211–1.0268)	2.4 (2.11–2.68)
4	4	9	1.0264 (1.0225–1.0302)	2.64 (2.25–3.02)	1.0242 (1.0214–1.0269)	2.42 (2.14–2.69)
4	4	10	1.0263 (1.0225–1.0302)	2.63 (2.25–3.02)	1.0244 (1.0216–1.0272)	2.44 (2.16–2.72)
4	4	11	1.0253 (1.0214–1.0293)	2.53 (2.14–2.93)	1.024 (1.0211–1.0268)	2.4 (2.11–2.68)

* P < 0.001.

RR, risk ratio; PC, percentage change; DF, degrees of freedom.

## Discussion

This study investigated the correlations between meteorological and environmental factors with the number of eczema outpatient visits in Guangzhou, a central city of south China located in the flourishing Pearl River Delta area. In total, 293,343 cases of eczema were analyzed using PM_2.5_ and PM_10_. The number of outpatient visits has been widely used in environmental epidemiology studies to estimate the influence of air pollutants on eczema. The confounders including seasonal changes and long-term trends can be modified using the distributed lag non-linear model (24–26). The obtained results suggested that the increasing concentrations of air pollutants were significantly associated with the rising number of eczema cases. The combined results of PM with other pollutants did not change significantly with PM alone. To date, no more research has revealed the effects of different combinations of pollutants on eczema. The effects of single pollutants on eczema still have different results in different regions, such as previous studies have revealed that O_3_ and NO_2_ were not associated with increased patient visits for AD but our team has revealed that NO_2_ exposure increases eczema outpatient (10, 27). The effect of pollutants on eczema is still inconclusive, thus, the effect of combined pollutants has not been concluded. Generally, the identification of vulnerable groups is of great significance for public health prevention. The existing evidence has limitations in revealing whether the associations of air pollution on eczema differ in population subgroups. Therefore, we stratified the eczema cases into three groups including <12, ≥12 and <65, and ≥65 years old. The obtained results indicated that children (<12) and the elderly (≥65) were more vulnerable to the adverse effects of short-term exposure to PM. The skin, which is the largest organ in the human body, contains four different barrier components including physical, chemical, microbial, and immunological barriers (28). The physical barrier, which mainly consists of the stratum corneum, can exert the protection function against the penetration of pathogens, allergens, and several other exogenous air pollutants such as PM, NO_2_, and SO_2_ (29, 30). A healthy skin barrier plays an essential role in preventing damage caused by stimuli including air pollutants. The vulnerable groups can easily suffer from eczema after short-term exposure to PM due to the immature and aging skin barrier in children and the elderly, respectively. Undeveloped sebaceous glands, skin infections, and local immune dysregulation are the main reasons for epidermal barrier abnormalities in children (31–33). On the other hand, epidermal dysfunction, compromised permeability homeostasis, reduced stratum corneum hydration, and elevated skin surface pH are the aggressive factors for the elderly (33, 34). Therefore, this study suggests that children and the elderly should avoid staying outdoors frequently to prevent PM exposure, which will potentially decrease the risk of eczema.

Previous studies conducted in Changsha, Shanghai, and Chengdu, representative cities with high urbanization in China, have reported that multiple air pollutants in both cities had the potential of increasing the incidence and prevalence of eczema (35–38). In Changsha, early childhood eczema is associated with exposure to air pollutants during both the preconceptional and perinatal periods (37, 38). In Chengdu, western China, the combined effect of NO_2_, SO_2_, and PM_10_ showed that the percentage change in daily outpatient visits for eczema increased (36). The Beijing study reported a significant positive correlation between the two pollutant models, but the impact was lower than the single pollutant model (39). The conclusions from both studies are consistent with the results obtained in this study. In addition, Dong et al. suggested that raising public awareness is crucial for constantly improving air quality. This is because PM is not only associated with cardiovascular and respiratory issues but also has a positive connection with the occurrence of skin diseases in China (40). Although a decreasing tendency in the levels of PM concentrations was observed from 2013 in the main regions in China including the Beijing-Tianjin-Hubei region, Yangtze River Delta, and Pearl River Delta, the recorded concentration levels still exceeded the limits recommended by the World Health Organization and Chinese authorities (41). With the acceleration of economic and industrial development, air pollution will most likely be an inevitable issue, particularly in developing countries. Although the increased risk we found is very small, the results of studies in some cities in China have found that the increase in PM will make AD more prone to the onset. If the concentration of PM can be reduced and the awareness of protection can be improved and promoted, it can prevent a large number of patients from relapse and shorten their sick time which will reduce a lot of health expenditure and burden.

However, the mechanisms of how PM induces and aggravates eczema in humans have not yet been elucidated. It is likely that PM induces the disruption of the epidermis by modulating the structural proteins including the small proline-rich (SPRR) family, occludens-1 (ZO-1), keratins, filaggrin, and claudin-1 (42–48), thereby resulting in both increased epithelial and endothelial barrier permeability (49). Moreover, a previous study reported that the penetration of PM was observed in both intact and barrier-disrupted skin *in vivo*, resulting in inflammatory responses (50). Current scientific opinions on the down-stream mechanisms through which air pollutants affect skin health mainly include: (1) elevation of oxidative stress *via* exogenous and endogenous reactive oxygen species (ROS) generation, which is a series of highly reactive chemical substances. Numerous pieces of evidence have suggested that PM can induce direct and indirect ROS formation, resulting in lipid, protein, and DNA damage (14, 51, 52); (2) activation of the aryl hydrocarbon receptor (AhR), which further limits cell proliferation and implicates in skin senescence (53); (3) activation of the inflammatory cascade in the skin and impairing of the immunological barrier (42, 46, 54); (4) activation of toll-like receptors, NF-κB, and MAPK signaling pathways (55–59); and (5) induction of apoptosis and autophagy (60, 61). In addition, SO_2_ can enhance the production of active oxygen and further reduce the content of antioxidants in the skin (62). Moreover, our previous study revealed that outpatient visits for patients with eczema were positively associated with short-term exposure to ambient NO_2_ (10). NO_2_ will damage the skin integrity which conducts a higher risk of exposure to allergic substances (63). Currently, the clinical management strategies for eczema mainly include the administration of moisturizers, topical corticosteroids, and calcineurin inhibitors, representing an anti-allergen and anti-inflammation strategy. In addition, to protect the skin, respiratory exposure should be noticed because respiratory exposure induces a higher risk of food allergy which will increase higher risk of eczema in children (64). Since the skin and reparatory is the most important defense barrier against environmental contaminants, future research should elucidate the clear mechanisms of how air pollutants aggravate eczema in humans with the overarching goal of providing recommendations for improving skin protection against air pollution.

To the best of our knowledge, this is the first study that has uncovered the associations and lagged effects between exposure to environmental factors and the number of eczema outpatients in Guangzhou, southern China. However, the study has several potential limitations. First, the study used data on the number of eczema outpatient visits to estimate the association of exposure to PM rather than using authentic physiological records or measurements. In addition, the clinical cases were only selected from one hospital. Although the hospital selection bias might be inevitable, the Dermatology Hospital of Southern Medical University is one of the largest and most representative dermatological departments among the local hospitals. Therefore, it can be used to reflect the trend of eczema outpatient visits from the whole region. Second, the data on the exposure of individuals to the environmental factors and the residual confounding factors (e.g., smoking, alcoholic consumption, dietary habits, and physical activities) were unavailable. Smoking, drinking, lack of exercise, etc. will lead to the recurrence of AD and prolonged attack time. Therefore, the lack of these data will make the data biased. Future research needs to collect more detailed personal data to avoid biased results. Furthermore, the outdoor average concentrations of PM_2.5_, PM_10_, NO_2_, SO_2_, and O_3_ were recorded as the average obtained from the different fixed monitoring stations, which may raise errors in documented measurements. Third, the study does not include detailed information on the severity of eczema and the differentiation of initial onset or relapsing cases, which may lead to bias while interpreting the results. The inaccurate grouping may result in biased results due to the severity and time of onset of the patient. Therefore, in the follow-up study, we will further take into account detailed information such as the age of onset of eczema, the time of onset, and the severity of the patient to further analyze from more dimensions. However, our study still offers considerable evidence on the relationship between environmental factors and the prevalence of eczema, thereby offering further motivations to investigate the mechanistic linkages between air pollutants and eczema, as well as proposing highlights for promoting the environmentally friendly actions which will lead to the reduction of air pollution for a better skin condition.

## Conclusion

This study has contributed to the limited scientific evidence which suggests that PM_2.5_ and PM_10_ may induce and aggravate eczema, especially among children and the elderly. The relationship between air quality trends and hospital resource arrangement should be paid attention to by hospital managers which may aid in disease prevention and lower the health burden.

## Data availability statement

The raw data supporting the conclusions of this article will be made available by the authors, without undue reservation.

## Ethics statement

The studies involving human participants were reviewed and approved by the Ethics Committee of Dermatology Hospital, Southern Medical University. Written informed consent to participate in this study was provided by the participants' legal guardian/next of kin.

## Author contributions

SS and JC designed the study. JZ, LF, and DJ analyzed the data. YY drafted the manuscript. BS and YC contributed to the data collection. All authors critically reviewed and approved the manuscript.

## Footnotes

^1^ http://sthjj.gz.gov.cn/zwgk/hjgb/content/post_5611849.html

## References

[1] Deckers I McLean S Linssen S Mommers M van Schayck C Sheikh A. Investigating international time trends in the incidence and prevalence of atopic eczema 1990–2010: a systematic review of epidemiological studies. PLoS ONE. (2012) 7:e39803. 10.1371/journal.pone.0039803 22808063 PMC3394782

[2] Nutten S. Atopic dermatitis: global epidemiology and risk factors. Ann Nutr Metab. (2015) 2015:8–16. 10.1159/000370220 25925336

[3] Karimkhani C Dellavalle R Coffeng L Flohr C Hay R Langan S. Global skin disease morbidity and mortality: an update from the global burden of disease study 2013. JAMA Dermatol. (2017) 153:406–12. 10.1001/jamadermatol.2016.5538 28249066 PMC5817488

[4] Behrendt H Alessandrini F Buters J Krämer U Koren H Ring J. Environmental pollution and allergy: historical aspects. Chem Immunol Allergy. (2014) 100:268–77. 10.1159/000359918 24925407

[5] Chong M Fonacier L. Treatment of eczema: corticosteroids and beyond. Clin Rev Allergy Immunol. (2016) 51:249–62. 10.1007/s12016-015-8486-7 25869743

[6] Blome C Radtke M Eissing L Augustin M. Quality of life in patients with atopic dermatitis: disease burden, measurement, and treatment benefit. Am J Clin Dermatol. (2016) 17:163–9. 10.1007/s40257-015-0171-3 26818063

[7] Chang W Lee C Hirota T Wang L Doi S Miyatake A. ORAI1 genetic polymorphisms associated with the susceptibility of atopic dermatitis in Japanese and Taiwanese populations. PLoS ONE. (2012) 7:e29387. 10.1371/journal.pone.0029387 22253717 PMC3258251

[8] Apfelbacher C Diepgen T Schmitt J. Determinants of eczema: population-based cross-sectional study in Germany. Allergy. (2011) 66:206–13. 10.1111/j.1398-9995.2010.02464.x 20804468

[9] Nikolopoulou M Kleissl J Linden P Lykoudis S. Pedestrians' perception of environmental stimuli through field surveys: focus on particulate pollution. Sci Total Environ. (2011) 409:2493–502. 10.1016/j.scitotenv.2011.02.002 21492905

[10] Zhang L Jing D Lu Q Shen S. NO exposure increases eczema outpatient visits in Guangzhou, China: an indication for hospital management. BMC Public Health. (2021) 21:506. 10.1186/s12889-021-10549-7 33722221 PMC7962398

[11] Kantor R Silverberg J. Environmental risk factors and their role in the management of atopic dermatitis. Expert Rev Clin Immunol. (2017) 13:15–26. 10.1080/1744666X.2016.1212660 27417220 PMC5216178

[12] Kabashima K Otsuka A Nomura T. Linking air pollution to atopic dermatitis. Nat Immunol. (2016) 18:5–6. 10.1038/ni.3615 27984566

[13] Kampa M Castanas E. Human health effects of air pollution. Environ Pollut. (2008) 151:362–7. 10.1016/j.envpol.2007.06.012 17646040

[14] Dijkhoff I Drasler B Karakocak B Petri-Fink A Valacchi G Eeman M. Impact of airborne particulate matter on skin: a systematic review from epidemiology to in vitro studies. Part Fibre Toxicol. (2020) 17:35. 10.1186/s12989-020-00366-y 32711561 PMC7382801

[15] Carlsten C Melén E. Air pollution, genetics, and allergy: an update. Curr Opin Allergy Clin Immunol. (2012) 12:455–60. 10.1097/ACI.0b013e328357cc55 22885891

[16] Oh I Lee J Ahn K Kim J Kim Y Sun Sim C. Association between particulate matter concentration and symptoms of atopic dermatitis in children living in an industrial urban area of South Korea. Environ Res. (2018) 160:462–8. 10.1016/j.envres.2017.10.030 29078139

[17] Ngoc L Park D Lee Y Lee Y. Systematic review and meta-analysis of human skin diseases due to particulate matter. Int J Environ Res Public Health. (2017) 14:1458. 10.3390/ijerph14121458 29186837 PMC5750877

[18] Guo P Wang Y Feng W Wu J Fu C Deng H. Ambient air pollution and risk for ischemic stroke: a short-term exposure assessment in south China. Int J Environ Res Public Health. (2017) 14:1091. 10.3390/ijerph14091091 28930181 PMC5615628

[19] Ravindra K Rattan P Mor S Aggarwal A. Generalized additive models: Building evidence of air pollution, climate change and human health. Environ Int. (2019) 132:104987. 10.1016/j.envint.2019.104987 31398655

[20] Schwartz J. Harvesting and long term exposure effects in the relation between air pollution and mortality. Am J Epidemiol. (2000) 151:440–8. 10.1093/oxfordjournals.aje.a010228 10707911

[21] Shen S Li X Yuan C Huang Q Liu D Ma S. Association of short-term exposure to sulfur dioxide and hospitalization for ischemic and hemorrhagic stroke in Guangzhou, China. BMC Public Health. (2020) 20:263. 10.1186/s12889-020-8354-0 32085727 PMC7035656

[22] Zhou M Wang L Liu T Zhang Y Lin H Luo Y. Health impact of the 2008 cold spell on mortality in subtropical China: the climate and health impact national assessment study (CHINAs). Environ Health. (2014) 13:60. 10.1186/1476-069X-13-60 25060645 PMC4115219

[23] Yin P Chen R Wang L Liu C Niu Y Wang W. The added effects of heatwaves on cause-specific mortality: a nationwide analysis in 272 Chinese cities. Environ Int. (2018) 121:898–905. 10.1016/j.envint.2018.10.016 30347372

[24] Bhaskaran K Gasparrini A Hajat S Smeeth L Armstrong B. Time series regression studies in environmental epidemiology. Int J Epidemiol. (2013) 42:1187–95. 10.1093/ije/dyt092 23760528 PMC3780998

[25] Dhadwal G Albrecht L Gniadecki R Poulin Y Yeung J Hong C. Approach to the assessment and management of adult patients with atopic dermatitis: a consensus document. Section IV: treatment options for the management of atopic dermatitis. J Cutan Med Surg. (2018) 22:21S–9S. 10.1177/1203475418805721 30439301

[26] Chow S Seow C Dizon M Godse K Foong H Chan V. A clinician's reference guide for the management of atopic dermatitis in Asians. Asia Pac Allergy. (2018) 8:e41. 10.5415/apallergy.2018.8.e41 30402408 PMC6209602

[27] Park T Park S Cho M Kim S. Associations of particulate matter with atopic dermatitis and chronic inflammatory skin diseases in South Korea. Clin Exp Dermatol. (2022) 47:325–34. 10.1111/ced.14910 34426985

[28] Grice E Segre J. The skin microbiome. Nat Rev Microbiol. (2011) 9:244–53. 10.1038/nrmicro2537 21407241 PMC3535073

[29] Scheuplein R Blank I. Permeability of the skin. Physiol Rev. (1971) 51:702–47. 10.1152/physrev.1971.51.4.702 4940637

[30] Madison K. Barrier function of the skin: “la raison d'être” of the epidermis. J Invest Dermatol. (2003) 121:231–41. 10.1046/j.1523-1747.2003.12359.x 12880413

[31] Gupta J Grube E Ericksen M Stevenson M Lucky A Sheth A. Intrinsically defective skin barrier function in children with atopic dermatitis correlates with disease severity. J Allergy Clin Immunol. (2008) 121:725–30.e2. 10.1016/j.jaci.2007.12.1161 18249438

[32] Cork M Danby S Vasilopoulos Y Hadgraft J Lane M Moustafa M. Epidermal barrier dysfunction in atopic dermatitis. J Invest Dermatol. (2009) 129:1892–908. 10.1038/jid.2009.133 19494826

[33] Ramos-e-Silva M Boza J Cestari T. Effects of age (neonates and elderly) on skin barrier function. Clin Dermatol. (2012) 30:274–6. 10.1016/j.clindermatol.2011.08.024 22507040

[34] Wang Z Man M Li T Elias P Mauro T. Aging-associated alterations in epidermal function and their clinical significance. Aging. (2020) 12:5551–65. 10.18632/aging.102946 32217811 PMC7138575

[35] Li Q Yang Y Chen R Kan H Song W Tan J. Ambient air pollution, meteorological factors and outpatient visits for eczema in Shanghai, China: a time-series analysis. Int J Environ Res Public Health. (2016) 13:e1106. 10.3390/ijerph13111106 27834842 PMC5129316

[36] Li A Fan L Xie L Ren Y Li L. Associations between air pollution, climate factors and outpatient visits for eczema in West China Hospital, Chengdu, south-western China: a time series analysis. J Eur Acad Dermatol Venereol. (2018) 32:486–94. 10.1111/jdv.14730 29194790

[37] Deng Q Lu C Li Y Sundell J Norbäck D. Exposure to outdoor air pollution during trimesters of pregnancy and childhood asthma, allergic rhinitis, and eczema. Environ Res. (2016) 150:119–27. 10.1016/j.envres.2016.05.050 27281689

[38] Lu C Deng L Ou C Yuan H Chen X Deng Q. Preconceptional and perinatal exposure to traffic-related air pollution and eczema in preschool children. J Dermatol Sci. (2017) 85:85–95. 10.1016/j.jdermsci.2016.11.004 27865567

[39] Guo Q Liang F Tian L Schikowski T Liu W Pan X. Ambient air pollution and the hospital outpatient visits for eczema and dermatitis in Beijing: a time-stratified case-crossover analysis. Environ Sci Process Impacts. (2019) 21:163–73. 10.1039/C8EM00494C 30632581

[40] Dong Y Liao L Li L Yi F Meng H He Y. Skin inflammation induced by ambient particulate matter in China. Sci Total Environ. (2019) 682:364–73. 10.1016/j.scitotenv.2019.05.155 31125750

[41] Zhang Z Wang J Hart JE Laden F Zhao C Li T. National scale spatiotemporal land-use regression model for PM_2.5_, PM_10_ and NO_2_ concentration in China. Atmos Environ. (2018) 192:48–54. 10.1016/j.atmosenv.2018.08.046

[42] Kim H Bae I Son E Park J Cha N Na H. Transcriptome analysis of airborne PM-induced detrimental effects on human keratinocytes. Toxicol Lett. (2017) 273:26–35. 10.1016/j.toxlet.2017.03.010 28341207

[43] Bae J Choi H Shin D Na H Park N Kim J. Fine particulate matter (PM_2.5_) inhibits ciliogenesis by increasing SPRR3 expression via c-Jun activation in RPE cells and skin keratinocytes. Sci Rep. (2019) 9:3994. 10.1038/s41598-019-40670-y 30850686 PMC6408442

[44] Lehmann A Blank F Baum O Gehr P Rothen-Rutishauser B. Diesel exhaust particles modulate the tight junction protein occludin in lung cells in vitro. Part Fibre Toxicol. (2009) 6:26. 10.1186/1743-8977-6-26 19814802 PMC2770470

[45] Pan T Wang P Aljuffali I Huang C Lee C Fang J. The impact of urban particulate pollution on skin barrier function and the subsequent drug absorption. J Dermatol Sci. (2015) 78:51–60. 10.1016/j.jdermsci.2015.01.011 25680853

[46] Jin S Li Z Choi E Lee S Kim Y Seo E. Urban particulate matter in air pollution penetrates into the barrier-disrupted skin and produces ROS-dependent cutaneous inflammatory response in vivo. J Dermatol Sci. (2018). 10.1016/j.jdermsci.2018.04.015 29731195

[47] Lee C Lin Z Hu S Chiang Y Hsu L Lin Y. Urban particulate matter down-regulates filaggrin via COX2 expression/PGE2 production leading to skin barrier dysfunction. Sci Rep. (2016) 6:27995. 10.1038/srep27995 27313009 PMC4911555

[48] Zhao R Guo Z Zhang R Deng C Xu J Dong W. Nasal epithelial barrier disruption by particulate matter ≤ 2.5 μm via tight junction protein degradation. J Appl Toxicol. (2018) 38:678–87. 10.1002/jat.3573 29235125

[49] Wang T Wang L Moreno-Vinasco L Lang G Siegler J Mathew B. Particulate matter air pollution disrupts endothelial cell barrier via calpain-mediated tight junction protein degradation. Part Fibre Toxicol. (2012) 9:35. 10.1186/1743-8977-9-35 22931549 PMC3489700

[50] Ali M Sayeski P Safavi A Lyles M Bernstein K. Janus kinase 2 (Jak2) must be catalytically active to associate with the AT1 receptor in response to angiotensin II. Biochem Biophys Res Commun. (1998) 249:672–7. 10.1006/bbrc.1998.9054 9731195

[51] Øvrevik J Refsnes M Låg M Holme J Schwarze P. Activation of Proinflammatory Responses in cells of the airway mucosa by particulate matter: oxidant- and non-oxidant-mediated triggering mechanisms. Biomolecules. (2015) 5:1399–440. 10.3390/biom5031399 26147224 PMC4598757

[52] Fang T Lakey P Weber R Shiraiwa M. Oxidative potential of particulate matter and generation of reactive oxygen species in epithelial lining fluid. Environ Sci Technol. (2019) 53:12784–92. 10.1021/acs.est.9b03823 31560535

[53] Ryu Y Kang K Piao M Ahn M Yi J Bossis G. Particulate matter-induced senescence of skin keratinocytes involves oxidative stress-dependent epigenetic modifications. Exp Mol Med. (2019) 51:1–14. 10.1038/s12276-019-0305-4 31551408 PMC6802667

[54] Ushio H Nohara K Fujimaki H. Effect of environmental pollutants on the production of pro-inflammatory cytokines by normal human dermal keratinocytes. Toxicol Lett. (1999) 105:17–24. 10.1016/S0378-4274(98)00379-8 10092052

[55] Kaisho T Akira S. Toll-like receptor function and signaling. J Allergy Clin Immunol. (2006) 117:979–87; quiz 88. 10.1016/j.jaci.2006.02.023 16675322

[56] Lebre M van der Aar A van Baarsen L van Capel T Schuitemaker J Kapsenberg M. Human keratinocytes express functional toll-like receptor 3, 4, 5, and 9. J Invest Dermatol. (2007) 127:331–41. 10.1038/sj.jid.5700530 17068485

[57] Liu T Zhang L Joo D Sun S. NF-κB signaling in inflammation. Signal Transd Targeted Therapy. (2017) 2:e23. 10.1038/sigtrans.2017.23 29158945 PMC5661633

[58] Smith W Garavito R DeWitt D. Prostaglandin endoperoxide H synthases (cyclooxygenases)-1 and−2. J Biol Chem. (1996) 271:33157–60. 10.1074/jbc.271.52.33157 8969167

[59] Kawai T Akira S TLR. signaling. Cell Death Differ. (2006) 13:816–25. 10.1038/sj.cdd.4401850 16410796

[60] Zhen A Piao M Hyun Y Kang K Madushan Fernando P Cho S. Diphlorethohydroxycarmalol attenuates fine particulate matter-induced subcellular skin dysfunction. Marine Drugs. (2019) 17:95. 10.3390/md17020095 30717280 PMC6410332

[61] Piao M Kang K Zhen A Fernando P Ahn M Koh Y. Particulate matter 2.5 mediates cutaneous cellular injury by inducing mitochondria-associated endoplasmic reticulum stress: protective effects of ginsenoside Rb1. Antioxidants. (2019) 8:e383. 10.3390/antiox8090383 31505827 PMC6769862

[62] Mancebo S Wang S. Recognizing the impact of ambient air pollution on skin health. J Eur Acad Dermatol Venereol. (2015) 29:2326–32. 10.1111/jdv.13250 26289769 PMC5916788

[63] Schikowski T Krutmann J. Air pollution (particulate matter and nitrogen dioxide) and skin aging. Hautarzt. (2019) 70:158–62. 10.1007/s00105-018-4338-8 30627745

[64] Zhang X Lu C Li Y Norbäck D Murthy P Sram R. Early-life exposure to air pollution associated with food allergy in children: Implications for 'one allergy' concept. Environ Res. (2023) 216:114713. 10.1016/j.envres.2022.114713 36347392

